# Adaptation of a web-based, open source electronic medical record system platform to support a large study of tuberculosis epidemiology

**DOI:** 10.1186/1472-6947-12-125

**Published:** 2012-11-07

**Authors:** Hamish SF Fraser, David Thomas, Juan Tomaylla, Nadia Garcia, Leonid Lecca, Megan Murray, Mercedes C Becerra

**Affiliations:** 1 Division of Global Health Equity, Brigham and Women’s Hospital, Boston, MA, USA; 2 Partners In Health, Boston, MA, USA; 3 Partners In Health, Lima, Peru; 4 Department of Global Health and Social Medicine, Harvard Medical School, Boston, MA, USA

**Keywords:** Electronic medical record, Developing country, Multidrug-resistant tuberculosis, MDR-TB

## Abstract

**Background:**

In 2006, we were funded by the US National Institutes of Health to implement a study of tuberculosis epidemiology in Peru. The study required a secure information system to manage data from a target goal of 16,000 subjects who needed to be followed for at least one year. With previous experience in the development and deployment of web-based medical record systems for TB treatment in Peru, we chose to use the OpenMRS open source electronic medical record system platform to develop the study information system. Supported by a core technical and management team and a large and growing worldwide community, OpenMRS is now being used in more than 40 developing countries. We adapted the OpenMRS platform to better support foreign languages. We added a new module to support double data entry, linkage to an existing laboratory information system, automatic upload of GPS data from handheld devices, and better security and auditing of data changes. We added new reports for study managers, and developed data extraction tools for research staff and statisticians. Further adaptation to handle direct entry of laboratory data occurred after the study was launched.

**Results:**

Data collection in the OpenMRS system began in September 2009. By August 2011 a total of 9,256 participants had been enrolled, 102,274 forms and 13,829 laboratory results had been entered, and there were 208 users. The system is now entirely supported by the Peruvian study staff and programmers.

**Conclusions:**

The information system served the study objectives well despite requiring some significant adaptations mid-stream. OpenMRS has more tools and capabilities than it did in 2008, and requires less adaptations for future projects. OpenMRS can be an effective research data system in resource poor environments, especially for organizations using or considering it for clinical care as well as research.

## Introduction

Peru has the highest incidence of tuberculosis (TB) in South America and the eighth highest burden of multidrug-resistant TB (MDR-TB) in the world [1]. Despite demonstrated success in the treatment of MDR-TB with an outpatient approach [2], it remains a problem in metropolitan Lima. Important questions remain about containing further spread of drug-resistant TB in high burden settings of this sort. In 2006, we were funded by the United States National Institutes of Health (NIH) to implement a study of tuberculosis epidemiology in Peru to determine mechanisms of MDR-TB transmission (Grant numbers: U01 AI057786, U19 AI076217). Implementing this study required a secure information system that would collect data from a target goal of 16,000 subjects, each followed for at least one year.

Traditionally, clinical research projects have built custom information systems for each study. Depending on the nature of the study, this approach can be expensive and time consuming, as each system needs to be meticulously tested and documented. In 2008, Lang et al. estimated that proprietary commercial systems for research data management can cost hundreds of thousands of dollars for a medium sized study, cost prohibitive in developing countries [3]. Projects that include longitudinal follow-up and multiple visits, forms, and laboratory results are particularly complex and expensive to support. Creating an effective information system to manage such data requires careful database design, and often necessitates significant adaptations to both design and software as new data items and workflows become apparent once the study has started.

Across study types there are common functional requirements for data collection systems; these include patient-based data collection, cohort analysis, scheduling of appointments or tests, patient identification using bar codes, linking to laboratory information systems, and ensuring security and confidentiality. Software tools created for these requirements can potentially be reused in new studies, reducing time, costs, and quality control issues. Recently, a number of clinical data management tools have become available in developed countries to standardize the management of clinical research data collection [4,5]. Research studies in developing countries face additional challenges in infrastructure, staffing and expertise, and are usually dependent on outside technical expertise and/or expensive software licenses for data collection systems. A similar situation exists with medical software systems in developing countries such as Electronic Medical Record (EMR), laboratory, and supply chain management systems [6].

When planning began for this study, we had extensive experience in developing information systems to support the clinical care of MDR-TB in Peru, including management of laboratory data [7]. We were keen to use a standard platform to develop the research information management system. In this paper we describe the customization and use of an open source EMR system platform called “OpenMRS” to support this large epidemiological study. We also describe the steps required to adapt and tailor the OpenMRS framework for this study, and our experience using the system for more than two years.

## Background

### The epidemiological study

The NIH funded the study (“Epidemiology of multidrug-resistant tuberculosis in Peru”) in 2006. This prospective study is designed to improve our understanding of the mechanisms of TB transmission as they relate to drug resistance and other microbial and host factors. It is referred to here by the name used by field staff: “Estudio Epi”. The primary study outcome measures are to identify (1) the relative risk of TB infection and disease in household members of TB patients who harbored drug-resistant *M tuberculosis* strains compared to household members of subjects with drug-sensitive strains, and (2) environmental and host factors associated with developing active MDR/XDR TB disease after exposure to an index case. Secondary study outcome measures are the rates of relapse and reinfection among TB patients. To meet the aims, the study team had to enroll and follow 4,000 TB patients and their household contacts. Sputum samples of those suspected to be infected were sent to a TB laboratory for smear and culture of *Mycobacterium tuberculosis* and drug sensitivity testing. Study subjects then needed to be followed for at least one year. A study field worker needed to complete baseline forms for each participant at a health center or home visit, and also record GPS coordinates of the home.

### Requirements for the system

Design of the information system began in January 2008 as the study protocol was being developed. The goals were to collect demographic and clinical data on 4,000 TB patients and their approximately 12,000 household members. We designed the system considering the following requirements:

• The system would be handling tens of thousands of study subjects and a large number of simultaneous users.

• Data would be collected on paper forms in the field, brought back to the study office, and double entered into the information system.

• Study workers would measure GPS coordinates at each subject’s home using hand-held devices containing lists of subjects, with automatic data upload.

• Local developers needed to be able to create multiple data entry forms in a standard manner.

• Reporting tools were needed to support the data center and study staff.

• Data would be de-identified and exported three times per week, and transferred to the research team servers in Boston.

• The system should be supported mostly or entirely by the Peruvian team.

#### Additional workflow and protocol requirements

Estudio Epi is bound by the requirements of the NIH approved protocol, which includes very strict rules determining who can view identified patient data. Field staff members visiting patients need to have access to names and addresses, but other staff carrying out data entry or other data management tasks should not be able to access identifying data. The protocol includes the use of barcodes for identifying each form and sample in order to speed up data entry and reduce errors. The use of barcodes also offers a reliable way to ensure a patient’s records are linked without the use of names.

The national TB guidelines in Peru called for the use of tuberculin skin testing in household contacts younger than 15 years old [8]. Because the study aimed to assess new infections in all the household contacts, our study protocol required the use of a tuberculin skin test in all these subjects, regardless of age. Because of this discrepancy between the national TB guidelines and our study protocol, the NIH classified the study as an interventional trial. The system was therefore required to meet US Food and Drug Administration (FDA) requirements for certification of software development processes, security of data, and auditing of any changes to data already entered (21 CFR 11 [9]). It also had to meet Good Clinical Practice requirements [10].

### OpenMRS

We used a software system called OpenMRS—the Open Medical Record System. OpenMRS was founded in 2005 as a collaboration to create common software tools to build EMR systems for use in developing countries [11]. This network has now been broadened to include many organizations in developing countries, with 21 countries represented at the OpenMRS annual meeting in Kigali, Rwanda in 2011. OpenMRS has been developed as open source software, and also supports open standards for the coding of medical data. OpenMRS is now used to support the care of patients in more than 40 developing countries [12,13], with versions to support the management of HIV and MDR-TB and, more recently, heart disease and primary care.

The design of OpenMRS offers a number of advantages for the development of research data management tools. Due to the focus on safe collection, storage, and management of clinical data, it includes auditing of data changes in the main database tables. This feature allows tracking of the history of changes in data items linked to the login of the user (although the original version of OpenMRS did not provide auditing of all required items for the study). There is a strong security system to ensure that only the authorized users have access to the clinical data, with the use of encryption of data transmitted over the web using Secure Socket Layer (SSL) Protocol [14]. OpenMRS is designed around a flexible data dictionary, called the concept dictionary, which allows new data items to be added without changing the underlying database structure. The dictionary simplifies the translation and maintenance of items in additional languages like Spanish. A major advantage of OpenMRS from a developer’s perspective is its modular software architecture. This allows separate components of software to “plug in” to the main system and allows additional functions to be added (and removed) without changing the core system (see glossary). OpenMRS is supported by a community of developers and implementers in many countries who communicate by email, regular conference calls, and periodic face-to-face meetings, with over 70 developers world wide contributing to the latest 1.9 release [12].

Using open source software (see glossary) for this project leveraged the design and programming of a much larger community than the specific developers working on Estudio Epi [12]. Most core features had already been built and tested, and new technical issues could be discussed with the community. The programming done for this project is being shared back with the OpenMRS community, a particularly important benefit in resource poor environments [3].

## Implementation

### Customization of OpenMRS for the study

The core OpenMRS software required limited changes; most additions described below were made in a separate OpenMRS software module.

#### Encoding of foreign languages

To handle the Spanish translation the core OpenMRS software required improvements to the system for supporting foreign language encoding (technically UTF-8 encoding) [15]. Improving this function also benefited translation to other languages used by the OpenMRS community.

#### Reconciliation of data entry discrepancies

At the time that we began to design this tailored system, OpenMRS routinely used a proprietary form-creation tool called Microsoft Infopath (Infopath, Microsoft, Inc., Redmond, WA). Double entry of data and reconciliation of errors was achieved by capturing Infopath data submissions and storing them in a holding table rather than allowing OpenMRS to process them immediately. This allows a senior data manager to review both entries and make corrections.

#### Linking to Global Positioning System (GPS) devices

Garmin devices (Garmin Inc. Olathe, KS) were used for GPS measurements. The company publishes an interface (technically a Javascript API) which was integrated into the Estudio Epi OpenMRS module. This integration allowed users to do two things: 1) download study patients to the Garmin handheld devices, and 2) automatically check for differences between the contents of the GPS device and the OpenMRS database and register new coordinate readings in the database. These actions occur when the user plugs in the GPS device to any data entry computer and navigates to the correct page using the web browser.

#### Links to existing laboratory data

Linking data from the public health laboratory was a priority for the study. The initial plan was to use the existing TB laboratory information system “eChasqui” developed by our team [16]; to implement this, we built a custom import module in OpenMRS. However, numerous organizational difficulties in the laboratories required a change in workflow. We subsequently designed a module to enter laboratory results into OpenMRS from paper forms.

#### Role-based security tools and applications

As required by the study protocol, we designed the information system such that the data entry staff never saw a participant’s name or address. The OpenMRS user privilege/role architecture was used to define which user could see what information; applications accessible were based on a user’s login. The data entry staff members never actually saw any of the “default” OpenMRS interface, just the components of the user interface for the Estudio Epi module that were allowable based on their specific privileges.

Auditing of changes in data entered was mainly achieved using the built-in tools in OpenMRS. Some additional tables had to be added to the database (using the study module) to track data items like patient encounters and some demographic data. This ensured that the system was compliant with FDA 21 CFR Part 11 [9].

#### Reporting tools and data export

Reporting is essential to assist study staff in ensuring that all data have been entered for each participant and in tracking potential data quality problems. We created a tailored reports module to allow the Peru-based programming team to program reports. These were mainly developed for the study coordinator and data managers to help administer the data entry process and to manage data updates.

The statistical analysis of the study data set required a full extraction of the data. The main tool for this purpose was written in SQL (a database language) and SAS (SAS Inc., Cary, NC) by the study staff. This tool is run on a de-identified version of the database that is automatically transferred to Boston three times a week. The end result of this process is a SAS-ready data set for analysis. This was the preferred approach by the statistical team; there are now tools in OpenMRS to automate many types of data export for users less experienced in programming.

### Infrastructure

IT infrastructure requirements were relatively simple, with a server accessed locally in the study office and over the Internet, standard Windows client computers, and bar code printers and readers for sites where patient registration occurred. Twenty Garmin GPS devices were purchased to track the location of each patient’s home and place of work. Data backups were performed nightly both on and off site. The information system was programmed by the lead developer (DT) who spent five months based in Lima, and trained three Peruvian programmers to develop and support the system during that period (including one of the authors, JT).

## Results

**Figure 1:**
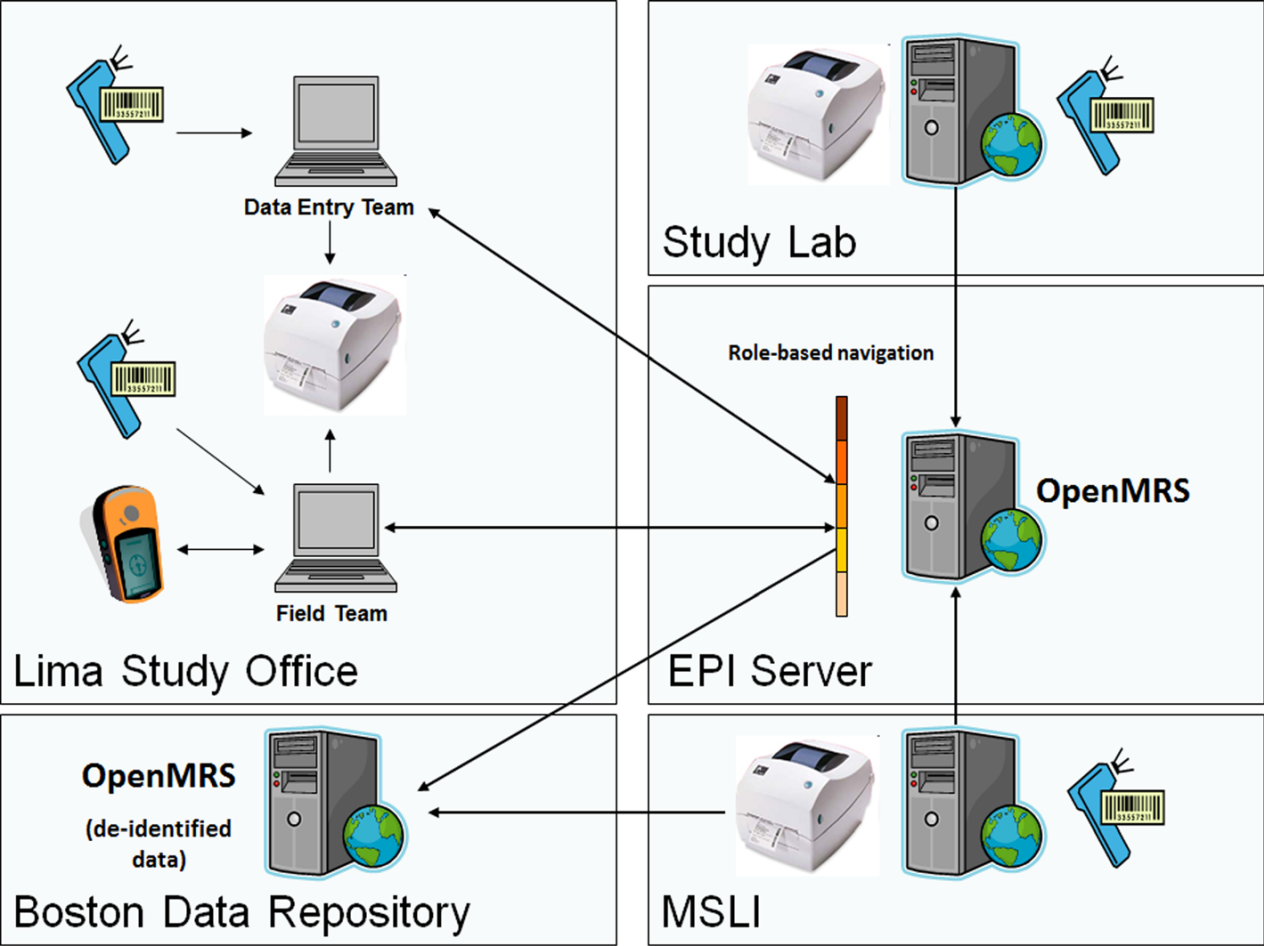
The work flow for data collection in the Estudio Epi System (MSLI stand for the Massachusetts State Laboratory Institute).

**Figure 2:**
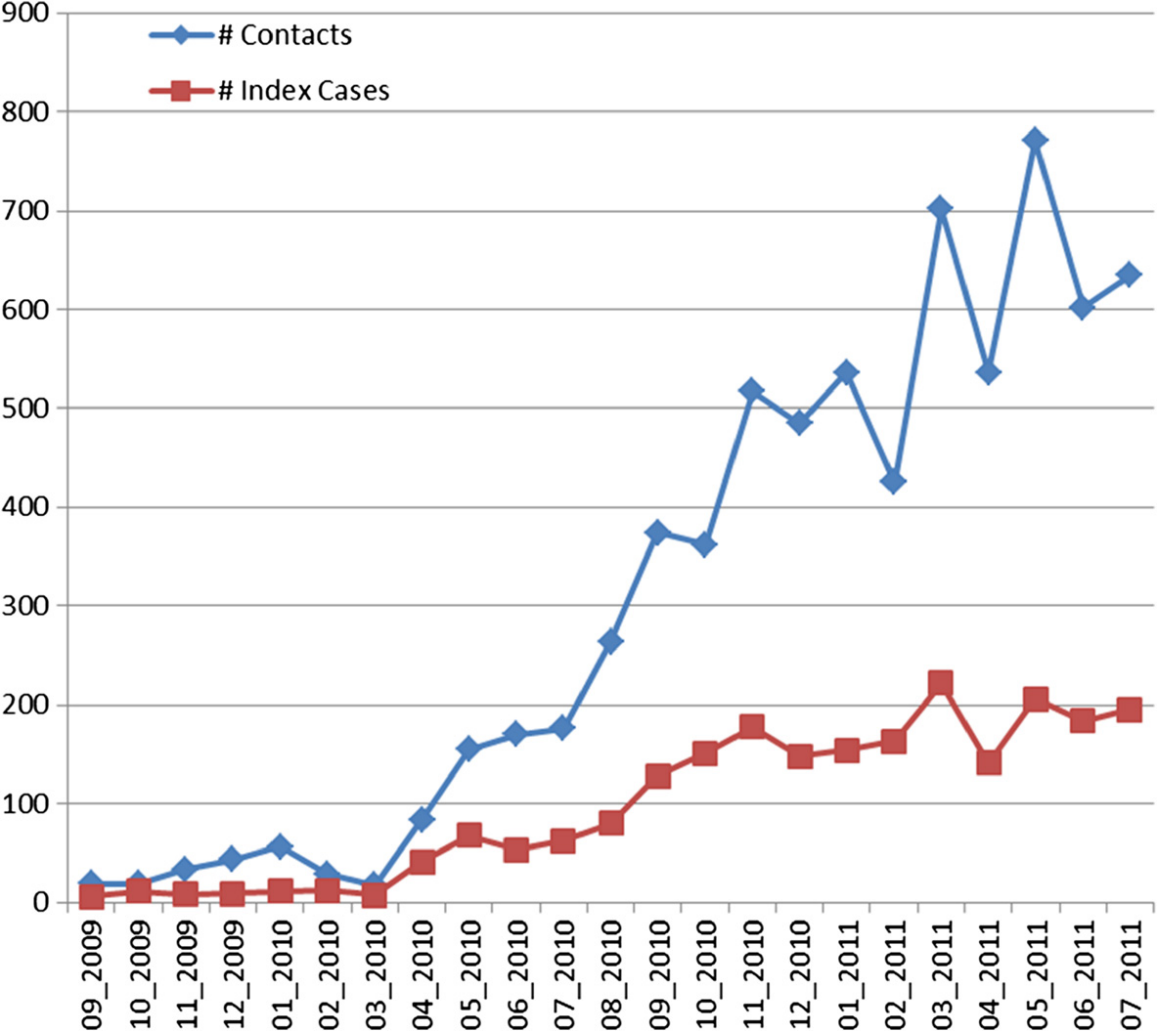
New patient entry per month in the OpenMRS system.

The OpenMRS system went live in September 2009. Figure 1 shows the structure and workflow of the system. By August 2011, the study team had used this system to collect data on one third of the projected number of participants; a total of 9,256 participants had been enrolled, of which 2,246 were TB patients and 7,010 were their household members. A total of 102,274 forms and 13,829 laboratory results had been entered, with an average of 1,197 forms or results entered per week. The data entry per month is shown in Figure 2.

**Figure 3:**
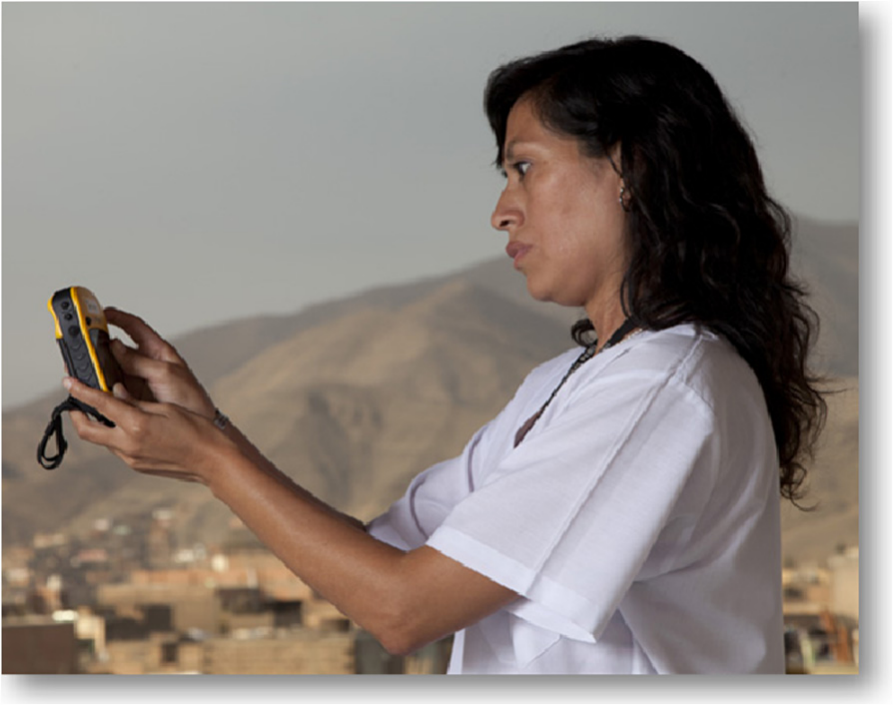
A field data collector taking GPS reading in northern Lima.

In August 2011 there were 208 direct users comprised of data management staff, field staff, laboratory staff, warehouse staff, and research staff. Figure 3 shows a field staff member taking a GPS reading. The system has 28 reports and 45 forms (33 using MS Infopath, with the rest programmed in HTML and Java). Users were trained by the core project team. Data management workers were trained for 40 hours and field workers (health center coordinators, laboratory workers and warehouse workers) were trained for 24 hours. Training manuals and documentation were developed for all users.

### Performance and support

Evaluation of the system was based on performance and reliability, as well as its ability to meet study requirements within time and cost constraints. The OpenMRS system launched on time and has run without significant breakdowns since then. Subsequent to the initial development period it required a further two months of support from the senior programmer. The Peruvian programmer worked full time during year one and half time in year two. Extra programming support was required to accommodate several changes in the study protocol that affected the processes for collecting and managing laboratory data as described in the methods. The main cost of implementing the software was the salaries for the programmers who added new functionality to the early version of OpenMRS; however, these adaptations have made the system more accessible for subsequent research studies, as well as more cost effective in the long term. A major workflow challenge identified was ensuring that data collection forms filled out by field-based staff were entered into the system in good time, this was a motivation to look at methods for direct field based data collection.

## Discussion

It is uncommon for a research project of this sort to use an Electronic Medical Record system platform to create the study database. This decision was motivated by the large size and complexity of the project, and by a desire to create stable and flexible tools that would be easily supportable. OpenMRS was able to be effectively tailored to the requirements of this study within the time frame required. The intention was also that the system could be reused for other studies within our organization and more broadly as part of our international development projects, a major benefit of open source software. In 2007 OpenMRS had already been tested in several countries with tens of thousands of patients, and was able to handle the large volume of data collected and managed in Estudio Epi without problems. Key benefits of using an existing software framework like OpenMRS include stability and reliability, availability of expertise and training materials, a wide range of existing functionality in software modules, support for a range of open standards for medical data coding and transmission, and a support network of programmers in our organization and the broader OpenMRS community. It can also result in a shorter time to create the system, especially if specialized functions are required as was the case for this study. Table 1 describes the pros and cons of using OpenMRS.

**Table 1:** Pros and cons of using OpenMRS for Estudio Epi

**Advantages**	**Disadvantages**
• Large user base and support team	• Workflow originally designed around clinical care rather than research
• Modular architecture simplifying the addition of new functionality and version control	• Required custom code for data exports to SAS
• Many modules and functions already built	• Required modification of form entry tools for double entry of data
• Support for open standards for data coding and exchange such as HL7, ICD [17], SNOMED [18], LOINC [19]	• Setup for a new study usually requires some programming
• Open source software	
• Good data security and auditing functions	
• Modules available to link to mobile phone software	
• Can be used online or offline	
• Potential to have major impact in resource poor environments given existing wide use for clinical care and reporting	

OpenMRS has also been used for research data collection in other projects in developing countries. Examples include the NIH funded IEDEA studies in East Africa, which collect data on HIV care in Kenya, Uganda and Tanzania and compare clinical outcomes [20]. The AMPATH project in Eldoret, Kenya has published multiple research studies based on data in OpenMRS from over 350,000 patient records (mostly HIV patients) collected over the last five years. Other uses include studies of the use and impact on clinical care of OpenMRS in Rwanda [21] and Kenya [22], and HIV clinical research studies in Peru and Rwanda. The Millennium Villages Project based at Columbia University is using OpenMRS for several studies on the impact of development strategies on health [23]. We expect many more projects to make use of data in their OpenMRS installations for clinical research [13].

### Problems and challenges

In order to create the database and system for this study, significant additions to OpenMRS were required initially, partly due to the relatively early stage of development of the overall OpenMRS system framework in 2007. The OpenMRS core worked well and has been successfully upgraded from version 1.4 to version 1.6.3. Almost all functionality was put into an OpenMRS module simplifying support and updates to the study specific enhancements. The lead Peruvian programmer (JT) continues to maintain the system and extends it to support new forms and reports. He identified several challenges with the system, including difficulties with special characters in Infopath forms, and the process of designing the forms to link to the concept dictionary. These issues can now be addressed more easily with the open source “HTML form-entry” module and a new reporting framework. Upgrading to new versions of OpenMRS can also require specialized support depending on which software modules are in use. A need for better technical documentation was also noted, and is being addressed with new training material and videos on the OpenMRS web site [24].

The biggest technical challenge in adapting and using OpenMRS relates to the data model based on the concept dictionary. Despite the many benefits of the design for data collection (as described above), such designs can make data extraction and use more complicated than a custom designed relational database. This complexity is partly due to the need to collect longitudinal clinical data with unspecified numbers of forms, laboratory results or other data etc. Creating the “flat” spreadsheet representations expected by statistical programs such as SAS (SAS inc., Carey, NC, USA) requires considerable transformation, particularly when the concept dictionary data model contains hierarchies that group together data items as OpenMRS does (for example to group results of drug sensitivity testing of mycobacterium by antibiotic type). A new OpenMRS module has recently been created to simplify the export of data sets for statistical analysis. This module creates one or more rows in a spreadsheet format for each instance of a particular form entered for a set of patients, and allows customization of which subjects and data items are exported. We had to create clear policies regarding who can see different types of data or groups of patients, and what tools and elements of data extraction tools should be available for whom and for what purposes. These rules, as well as the work flow and software, had to be updated after the commencement of the study but are proving useful in other PIH projects.

A number of problems have to be overcome to achieve effective data collection and management in resource poor environments [13,25]. These include:

• Infrastructure including hardware, power, networking and often internet access.

• Staff to carry out data entry, data management and quality control, IT support and software support.

• Training for the core staff and other users of the system.

• Leadership and management to ensure that the key aspects of the system are prioritized and supported and problems addressed promptly.

• Software.

Considering the interlinked nature of these five requirements, it is essential for all five to be fulfilled in order for the project to succeed. In developing countries it is particularly important to have software that is stable, easily updated, supported locally and well documented. It is also beneficial to have more than one programmer who knows the system and who can develop local programming and IT capacity. Too often projects rely on foreign technical assistance which can cause long delays and is expensive, especially for projects that are not funded with external grants.

Many research data management systems are dependent on reliable internet access. Internet was available in Lima, but is a major challenge in other parts of Peru and in most developing countries. We needed to use local servers for rapid response and to ensure patient-identifiable data remained in the country. In PIH-supported clinical care projects in Rwanda we have developed a system for intermittent synchronization of data between clinical sites and a master server, which could potentially be used for research studies in sites with unreliable internet.

### Alternative research data management systems

At the start of Estudio Epi there were few suitable options available for research data management [3]. There are now potential alternative open source or shared source research data management systems. Leroux and colleagues examined major commercial and open source clinical trial management systems and developed a checklist of key characteristics required for their projects shown in Table 2[26]. They identified OpenClinica [27] as best fitting their criteria. OpenClinica is a flexible web-based open source system for clinical data management and has been used for many clinical trials over the last three years. OpenMRS was not considered for that study, but the version described here for Estudio Epi fits ten of the eleven criteria. The exception is support for the Clinical Data Interchange Standards Consortium (CDISC); however, OpenMRS does support a range of clinical data exchange standards.

**Table 2:** **Checklist of desirable features for research data management systems**[26]

1	Implement security measures and protocols that prohibit unauthorised access to the study and data.
2	Provide adequate audit trail to ensure that all changes pertaining to the conduct of the trial are well documented.
3	Incorporate features to encourage the consistent use of clinical terminology and to alert users that data is out of range.
4	Provide suitable safeguards to isolate identifiable information from the study and ensure that retrieved data regarding each subject is only attributable to that subject.
5	Provide satisfactory backup and recovery protocols to guard against data loss.
6	Provide support for several types of fields (such as dates, text, numerical values) and in various formats (such as files, x-ray images).
7	Facilitate data extraction and the ability to swiftly generate reports.
8	Uphold the cost effectiveness of the system.
9	Endorse minimal development efforts
10	Advocate an advantageous type of licensing.
11	Promote adherence to industry standards, such as the Clinical Data Interchange Standards Consortium (CDISC)

Another system with increasingly wide use is Project REDCap, a web-based clinical trial management system developed at Vanderbilt University [5]. It was started in 2004 as a way to create forms and other tools for clinical data management that do not require programming or creation from scratch in a database management system like MS Access. The system is “freeware” in that it can be used on the web or installed on a local server freely and with new features added, but the ownership of the software remains with Vanderbilt University. At present OpenClinica and REDCap do not support offline data entry, requiring either good internet connections or well supported local installations. OpenCDMS is another open source web-based research data management system. It was developed at Manchester University, UK [28] and provides a wide range of functions for clinical trial management. There is also a Peruvian developed, web-based “computer assisted self interviewing” system called SISQUAL used for HIV research interviews. Like OpenClinica, OpenCDMS and SISQUAL are 21CFR Part 11 compliant. These systems provide good tools for many research studies, but in comparison to OpenMRS they are not designed for clinical care data, and lack the support for certain open medical data standards. In the case of REDCap, the code is not truly open source, so users or countries do not have full control of their installations and improvements.

## Conclusions

As the scale of the Estudio Epi study grew it became clear that the workflow and productivity of field staff would benefit greatly from the ability to enter data directly into mobile devices such as cell phones or PDAs, an approach we had previously validated with laboratory data in Lima [29]. Work is currently ongoing to add that capability with an open source software system called OpenXdata [30]. In 2012 there will be improved integration of OpenMRS with mobile software tools through better support for web services. This integration should improve interoperability with a range of mobile phone based data collection tools including Open Data Kit [31], Comcare [32], Sana [33], and Epi-surveyor [34].

Another area of active development for OpenMRS is improved interoperability with other health information systems, including those for managing laboratory, pharmacy, and genomic data. It is now possible to export data from OpenMRS to i2b2, an open source system that allows users to create queries across multiple clinical and research databases such as EMRs and genomic databases [35]. Data export between OpenMRS and i2b2 can assist in the initial research to identify interesting subject groups. The data warehouse system Pentaho [36] is also being integrated with OpenMRS to allow a wide range of analyses and data exports. The concept dictionary allows sharing of data dictionaries between OpenMRS systems facilitating standardization of data collection and analysis. Tools are now being developed to create such core data dictionaries and share them for clinical and research purposes, including maternal health care initiatives. Research on data collected for clinical purposes in EMR systems is an important priority for many organizations, and OpenMRS is uniquely positioned to support such work especially in resource poor environments.

Developing countries are increasingly implementing policies about who can access and use clinical data for research purposes. The tools and experience gained from the adaptation of OpenMRS for Estudio Epi will help improve the management of data in accordance with these rules in other countries, such as in Rwanda where PIH is currently rolling out this system for a large clinical research study.

The choice of systems for research data collection is now growing with good options for stand-alone clinical studies in sites with reliable internet access. Our experience and that of other users suggests that OpenMRS has a valuable role for research data collection in resource poor environments, especially if there is a need to collect data for both clinical and research purposes, or to link to data sources such as laboratories using coding and data exchange standards. Our team and the OpenMRS community are continuing to improve the tools available. With an installed base in more than forty developing countries, there is great potential for OpenMRS to improve support for clinical research either alone or in combination with other systems.

## Availability and requirements

All software, source code, documentation and training materials is available for download at http://www.openmrs.org under an open source or creative commons license.

## Consent

Written consent was obtained from the person pictured for the publication of the photograph in Figure 3. A copy of the written consent is available for review by the Editor of this journal.

## Abbreviations

Information system: used here to refer to the software to manage data. It can also be used to describe the broader aspects of information collection, flow and use.; Database: a piece of software that is used to store and retrieve data. It is part of the software of an information system.; Open source software: software that is freely shared with all users including the underlying source code that is written by the programmers. Users can examine and modify the code but have to share improvements with the broader community. It includes an open source license which specifies the extent of the code that must be shared and is hosted on a web site that allows downloads of the source code plus documentation and often full working versions. If these rules are not strictly observed organizations may not be confident that they will always have access to the source code.; 21 CFR Part 11: US federal government regulations governing the design, programming, testing and use of software for managing clinical research data. They apply to studies that are regulated by the FDA such as certain clinical trials. A particular focus is the auditing of any changes in the data collected such as edits and who made them to ensure data integrity [9].; Good Clinical Practice requirements: a set of internationally recognized quality standards for clinical research [10].; Electronic Medical Record or EMR: a software program that allows the collection, storage, display and analysis of individual patient records primarily to support clinical care. Such data may also be used for clinical research such as carrying out queries for groups of patients with certain characteristics for further study.; Secure Socket Layer or SSL: a software protocol that is used to ensure that data between a web server and web browser is encrypted to prevent third parties from viewing or modifying it. It is typically used for financial transactions on the web as well as to ensure confidentially of personal records.; OpenMRS modular architecture: OpenMRS is designed to all additional software components to be added as plugins without changing the underlying core software. This ensures that projects can benefit from the stable, well tested core code while being able to innovate and make improvements to the system. Some modules are used by most implementations and are considered part of the main OpenMRS system, while others are very specific to particular projects.

## Competing interests

The authors declare they have no competing interests.

## Authors’ contributions

HF conceived of the original plan to use OpenMRS, oversaw the initial stages of the work and led the writing of the paper. DT designed the software modifications for OpenMRS and with JT programmed and implemented the system. He also co-wrote the paper. JT programmed substantial parts of the system, and supported the system on a daily basis. NG designed much of the workflow for the cohort study and co-designed the workflow of the information system. LL led the cohort study in Peru and oversaw the development of the protocols implemented by the information system. MM conceived of the cohort study and provided overall leadership. MB conceived of the cohort study and provided overall leadership, and co-wrote the paper. All authors provided critical comments and ideas for the paper and read the final draft. All authors read and approved the final manuscript.

## Authors’ information

HF is an assistant professor at Harvard Medical School, a research associate at the Brigham and Women’s Hospital, and a co-founder of the OpenMRS project.

DT is a Java programmer and public health specialist with a focus on medical information systems.

JT is a Java programmer at Partners In Health in Peru.

NG is a nurse and study manager at Partners In Health in Peru.

LL is a senior TB specialist at Partners In Health and the local PI of the cohort study.

MM is a professor at Harvard Medical School, Co-PI of the cohort study, and PI of the U19 award.

MB is an epidemiologist and associate professor at Harvard Medical School, Co-PI of the cohort study, and PI of the U01 award.

## Pre-publication history

The pre-publication history for this paper can be accessed here:

http://www.biomedcentral.com/1472-6947/12/125/prepub
